# MLPH regulates EMT in pancreatic adenocarcinoma through the PI3K-AKT signaling pathway

**DOI:** 10.7150/jca.94573

**Published:** 2024-09-09

**Authors:** Mengda Wei, Xi Yang, Xiaoying Yang, Yanqing Huang, Zhenmin Yuan, Junjie Huang, Junren Wei, Lei Tian

**Affiliations:** 1 Department of Gastrointestinal Surgery, The First Affiliated Hospital of Guangxi Medical University, Nanning, China.; 2 Guangxi Key Laboratory of Enhanced Recovery After Surgery for Gastrointestinal Cancer, Nanning, China.

**Keywords:** MLPH, epithelial-mesenchymal transition, pancreatic adenocarcinoma, PI3K-AKT signaling pathway, progression

## Abstract

Pancreatic adenocarcinoma (PAAD) is an extremely malignant tumor, and most patients develop postoperative metastases. Melanophilin (MLPH) is involved in the progression of various tumors, but its molecular mechanisms and role in pancreatic cancer progression are unknown. In this study, differential MLPH expression in cancer tissues and the adjacent tissues was evaluated using the Gene Expression Profiling Interaction Analysis 2 (GEPIA 2) and Human Protein Atlas (HPA) databases. The role of MLPH in PAAD proliferation, invasion, and migration *in vitro* was explored *via* clone formation, Cell Counting Kit-8 assay, Transwell assay, and western blot. The *in vivo* validation of function was performed using a metastatic nude mouse model. The result showed that the pancreatic cancer tissues had significantly higher MLPH expression levels than the noncancerous pancreatic tissues. MLPH expression changes were related to PAAD cell proliferation, invasion, and migration. The western blotting demonstrated that PAAD cells had reduced Epithelial-mesenchymal transition (EMT)-related marker expression. Furthermore, overexpressing MLPH enhanced cell proliferation, migration, and invasion, and increased EMT-related marker expression. The Kyoto Encyclopedia of Genes and Genomes (KEGG) enrichment analysis revealed that the molecular mechanism underlying the effect of MLPH on PAAD was significantly related to the PI3K-AKT pathway. LY294002 blocked the MLPH overexpression-mediated enhanced cell invasion and migration and inhibited EMT-associated marker expression. Conversely, 740Y-P reversed the inhibitory effects of MLPH downregulation and led to cell migration, invasion, and EMT. MLPH regulated EMT to mediate PAAD cell invasive migration through the PI3K-AKT pathway. The results indicated that MLPH is a possible target for blocking PAAD metastasis.

## Introduction

Pancreatic adenocarcinoma (PAAD) is commonly diagnosed globally. It has become the third leading cause of death related to cancer 1 and has a 5-year survival rate of only 10% 2. Only 15% of patients with PAAD have access to surgery, and >60% of patients who had undergone surgery developed distant metastases within 24 months after surgery^3^. The median postoperative patient survival time is only 20-23 months^4^. Therefore, there is an urgent need for more effective interventions against tumor invasive migration.

A number of reports have shown that Epithelial-mesenchymal transition (EMT) is closely linked to PAAD invasion and migration progression^5^, ^6^. EMT is the physiological procedure of embryonic development and tissue remodeling. EMT is involved in the progression of several malignancies, where epithelial cells lose their polarizing properties and stable cell adhesion, and then acquire a mesenchymal cell phenotype^7^, ^8^. Cells that have undergone EMT have downregulated the expression level of E-cadherin and upregulated the expression levels of N-cadherin, Slug, vimentin^9^, ^10^. Tumor cells acquire stronger invasive and migratory properties via EMT, and proliferate and metastasize after extravasation^11^. Thus, regulating EMT is crucial to control pancreatic cancer cell migration and invasion.

Melanophilin (MLPH) is a protein associated with melanosome delivery, and it has been extensively studied in animal hair pigmentation^12^, ^13^ and Griscelli syndrome type 3, which stems from MLPH gene deficiency in humans^14^, ^15^. Recently, MLPH has been shown to correlates with the progression of various tumors, and has become a prognostic indicator and treatment target for a variety of tumors, such as Spitz tumor^16^, melanoma 17, small cell lung cancer^18^, bladder cancer 19, prostate cancer^20^, breast cancer^21^, and rectal cancer^22^. High MLPH expression in patients with rectal cancer has exhibited association with poorer preoperative radiotherapy response and lower survival 22. Furthermore, in prostate cancer studies, MLPH influences tumor progression by upregulating EMT 23. In conclusion, MLPH can influence tumor prognosis and regulate EMT to affect tumor invasion and migratory progression.

In cancer, the PI3K-AKT signaling pathway sustains tumor cell biological properties^24^, ^25^ and stimulates tumor cell metastasis and invasion by prompting EMT directly or through synergistic induction with other signaling pathways 26, 27. PSCs induce EMT and inhibit the apoptosis of cancer cells via paracrine activation of the PI3K-AKT signaling pathway, thereby enhancing pancreatic cancer cell chemoresistance 28. Similarly, curcumin impedes EMT induced by superoxide dismutase through the PI3K-AKT-NF-κB pathway 29. FAM126A interacts with ENO1 to mediate pancreatic cancer cell metastasis and proliferation via the PI3K-AKT signaling pathway^30^. These studies confirmed the importance of the PI3K-AKT pathway in mediating EMT to promote PAAD cell invasion and migration.

As mentioned earlier, MLPH is also likely to promote PAAD cell invasion and migration by activating the PI3K signaling pathway to regulate EMT. However, there are no studies on the specific mechanism and role of MLPH in PAAD. Accordingly, to explore whether MLPH mediates EMT regulated PAAD cell invasion and migration through the PI3K-AKT signaling pathway, we performed bioinformatics, cell function experiments, and western blotting experiments. These experiments enable us to further understand the mechanism by which MLPH affects PAAD progression and to provide a research basis for MLPH as a potential prognostic factor and therapeutic target for PAAD.

## Material and Methods

### Cancer Genome Atlas (TCGA) data acquisition and analysis

PAAD RNA sequencing expression data (fragments per kilobase per million mapped fragments format) were acquired from TCGA database (https://portal.gdc.cancer.gov/). The dataset contained 183 PAAD samples (179 pancreatic cancer samples and 4 adjacent normal tissue samples). The detailed criteria for the inclusion of patients enrolled in the study were as follows: 1. Pathologic confirmation: A pathologically confirmed diagnosis of pancreatic cancer. The criteria for exclusion were as follows: 1. Unclear pathological diagnosis: Unclear pathological diagnosis or non-pancreatic cancer. 2. Benign pancreatic diseases: Such as chronic pancreatitis, pancreatic cysts, and other benign diseases. 3. Other pancreatic lesions: Such as pancreatic islet cell tumors, pancreatic endocrine tumors, and so on. 4. Other systemic diseases: Systemic diseases that may affect the expression of pancreatic cancer-specific genes or pathological features. MLPH expression differences in the samples were examined using a nonparametric test or an unpaired t-test. Gene expression data were stored for bioinformatics analysis.

### Gene Expression Profiling Interaction Analysis 2 (GEPIA 2) database analysis

MLPH expression in PAAD and other cancer types was examined using the GEPIA 2 database (http://gepia2.cancer-pku.cn/#index). The GEPIA 2 database combines TCGA and Genotype-Tissue Expression (GTEx) gene expression data.

### Human Protein Atlas (HPA) database analysis

Protein expression profiles in human tumor tissues were evaluated using the HPA public database (https://www.proteinatlas.org/). MLPH protein expression levels in noncancerous pancreas and PAAD tissue in the HPA database were explored.

### Kaplan-Meier Plotter database analysis

The correlation between MLPH expression and PAAD prognosis was assessed in the Kaplan-Meier Plotter database (http://kmplot.com/analysis/) based on the TCGA-PAAD dataset.

### Correlation analysis of MLPH expression and clinical characteristics

Expression data and clinical data of the samples were extracted from TCGA-PAAD, normal and non-clinical samples were removed, statistics were performed using the R package 'stats' and the 'car', and the data were visualized using the 'ggplot2'package.

### Kyoto Encyclopedia of Genes and Genomes (KEGG) pathway analysis

The biological pathways containing enriched genes were searched using KEGG analysis. The 179 pancreatic cancer samples were classified into high and low expression groups based on the median expression value of MLPH, and the DEGs (|logFC| > 1, FDR < 0.05) were analyzed using the R package 'limma'. The KEGG pathways enrichment analysis (*P* < 0.05) was performed using the R packages 'clusterProfiler' and 'enrichplot' and the results were visualized using the R package 'ggplot2'.

### Cell culture and reagents

The human pancreatic cancer cell line PANC-1 was from Pricella and SUIT-2 human pancreatic cancer cells were from CellCook. The PANC-1 cells were cultured in high-glucose Dulbecco's modified Eagle's medium (DMEM: Gibco) supplemented with 10% fetal bovine serum (FBS: VivaCell) and 1% penicillin-streptomycin (P/S: Biosharp). We cultured the SUIT-2 cells in minimum essential medium (MEM: Gibco) containing 10% FBS,1% P/S, and 1% MEM non-essential amino acids. The cells were incubated with 5% CO^2^ at 37°C. The PI3K-AKT pathway was inhibited using 1μM LY294002(MCE) and activated using 30μM 740Y-P (MCE).

### Small interfering RNA (siRNA) transfection

The cells were cultured to 50% confluence in 6-well plates. Opti-MEM (Gibco) basal medium was mixed with Hieff Trans transfection reagent (Yeasen) and 50nM MLPH siRNA (si-MLPH: Guangzhou RiboBio Biotechnology Co., Ltd.), incubated for 10 min at room temperature, then added to the 6-well plates. The complete medium was replaced with fresh complete medium after 24 h. The sequences of siRNA are as follows: GGAAGTTGTTCAACGAGAT.

### Plasmid transfection

The LV-oe-MLPH (human)-3*Flag-Puro overexpression plasmid was constructed by MiaoLingBio (MiaoLingBio, China). The overexpression plasmid, Hieff Trans, and Opti-MEM were mixed according to the reagent instructions and incubated for 5 min at room temperature. Then, the mixture was added to the 6-well plates. After 4-6 h, the medium was replaced with complete medium.

### Lentivirus transfection

MLPH stable knockdown cell lines were constructed by lentiviral transfection, and cells in the logarithmic growth phase were transfected when the cell density reached 90%, puromycin (2 mg/ ml) was added for screening, and stable cell colonies were expanded after a period of 10-14 days.

### Quantitative real-time PCR

Total RNA was isolated using NucleoZOL (Macherey-Nagel GmbH & Co. KG) reagent. We synthesized complementary DNA (cDNA) according to the reverse transcription kit (Vazyme) instructions. Real-time quantitative PCR was conducted using the Applied Biosystems™ 7500 (ThermoFisher) unit according to the SYBR Green Chimeric Fluorimetry Kit (Vazyme) instructions. The primer sequences are as follows:

MLPH-F: TGCTTGCCCCCATTATCCAG

MLPH-R: CTTGCCCTTCAACGCCTCTA

ACTB-F: TGGCACCCAGCACAATGAA

ACTB-R: CTAAGTCATAGTCCGCCTAGAAGCA

### Western blot assay

We separated equal amounts of protein from whole-cell lysates with sodium dodecyl sulfate-polyacrylamide gel electrophoresis (SDS-PAGE), then transferred to PVDF membranes. The membranes were incubated with the primary antibodies (Cell Signaling Technology: E-cadherin,1:1000; N-cadherin, 1: 1000; Phospho-Akt, 1:1000; Akt, 1:1000. Proteintech: MLPH, 1:1000; Vimentin, 1:1000; β-actin, 1:10000) overnight at 4°C. Then, the membranes were incubated with the secondary antibody for 1 h at room temperature. Finally, the blots were exposed using the Fluor-Chem M imaging system.

### Transwell assay

Transwell chambers were used to conduct the assays on cell migration. Cells (3 × 10^4^) were suspended in 200μL serum-free medium and inoculated into the upper chamber. The lower chamber contained complete medium (700 µL) as an attractant. After 24-48-h incubation, the cells in the upper chamber were collected using cotton swabs and fixed in 4% paraformaldehyde. Then, the cells were stained with 500 µL crystal violet dye solution for 20 min, washed a minimum of three times with phosphate-buffered saline (PBS), and air-dried. Subsequently, the cells were counted.

The invasion assay was performed using the same procedure as the migration assay. However, the invasion assay involved coating the upper Transwell chamber with Matrigel, whereas the migration experiment did not.

### Colony formation assay

PANC-1 or SUIT-2 cells (1 × 10^3^) were added to 35-mm culture dishes. After 10-14-day incubation, the medium was removed, and the cells washed gently with PBS three times, then fixed in 4% paraformaldehyde. Next, the cells were stained with crystal violet dye solution (500μL) for 20 min, washed a minimum of three times with PBS, air-dried, and photographed.

### Cell Counting Kit-8 (CCK-8) assay

We seeded the transfected PANC-1 cells and SUIT-2 cells in 96-well plates. Complete medium (100 µL) with 10 μL CCK-8 reagent was added to each well at five time points and incubated for 1 h in a 37°C CO^2^ incubator. The cell absorbance at 450 nm wavelength was detected.

### *In vivo* experiment

The study was approved by the Medical Ethics Committee of the First Affiliated Hospital of Guangxi Medical University (No. 2023-S957-01). Four-week-old female nude mice (BALB/c-nu) were purchased from the Experimental Animal Center of Guangxi Medical University and were kept under pathogen-free (SPF) rearing conditions in the Experimental Animal Center of Guangxi Medical University. si-MLPH-treated SUIT-2 cells and normal SUIT-2 cells (5×10^6^) were injected subcutaneously into the mice(n=5). The LV-Vector-transfected SUIT-2 cells and LV-oe-MLPH-transfected SUIT-2 cells (5×10^6^) were injected subcutaneously into the mice(n=5). Tumor volume was detected after 14 days, calculated as V=π/6*L*W^2^, where "L" and "W" represent the maximum and minimum diameters of the tumor, respectively.

To thoroughly validate the impact of MLPH on tumor metastasis *in vivo*, we constructed a nude mouse tail vein lung metastasis model. The cells were injected into nude mice through the tail vein. The mouse lung tissue was removed and metastatic nodules were counted after 4 weeks.

### Statistical analysis

All data were analyzed using GraphPad 8.0 statistical software. The means ± standard deviations were reported. Two groups were compared using a t-test. Multiple groups were compared using one-way analysis of variance. P < 0.05 was considered significant, and the results were: * *P* < 0.05, ** *P* < 0.01, *** *P* < 0.001.

## Results

### MLPH expression was increased in PAAD

**Figure 1:**
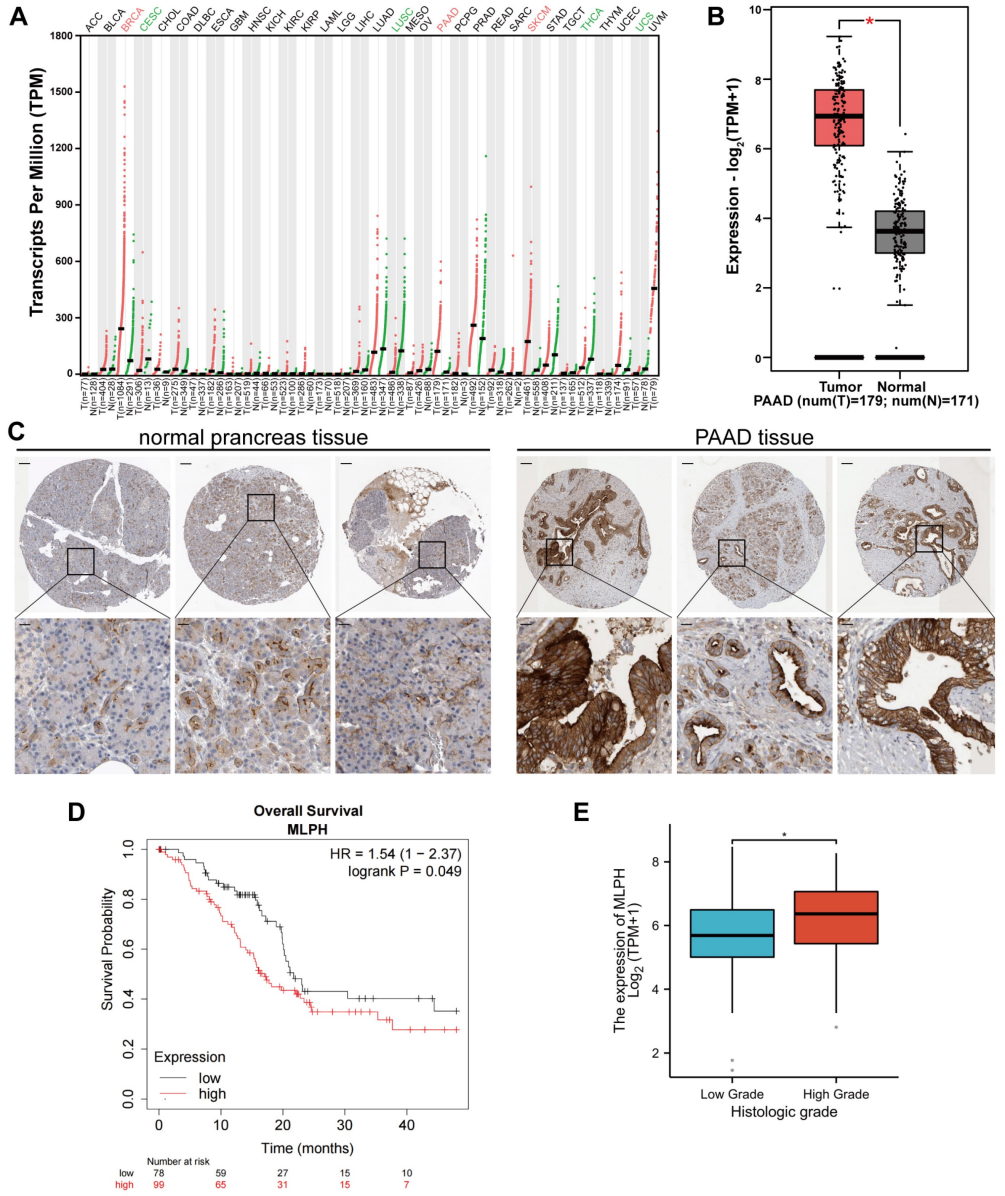
** Differential expression of MLPH.** (A)According to the GEPIA 2 database, MLPH is highly expressed in several cancer tissues including PAAD. (B) MLPH is highly expressed in PAAD tissues. (C) Representative immunohistochemical staining images of MLPH in PAAD and normal tissues from the HPA database. (D) Overall Survival analysis in the Kaplan-Meier Plotter database based on TCGA data. (E) MLPH mRNA expression level in different Histologic Grade of PAAD samples. (The scale bar is 200 μm, the locally enlarged scale bar is 50 μm.*P<0.05).

The GEPIA 2 database analysis demonstrated that MLPH expression was upregulated in breast invasive carcinoma, PAAD, prostate adenocarcinoma, and skin melanoma. Contrastingly, MLPH expression was downregulated in cervical cancer, lung squamous carcinoma, thyroid cancer, and uterine sarcoma (Figure 1A). GEPIA 2 analysis of TCGA and GTEx gene expression data revealed that PAAD tissues had higher MLPH expression than the noncancerous pancreatic tissues (Figure 1B). We also analyzed the HPA immunohistochemical samples and verified that the PAAD tissues had higher MLPH expression levels than the noncancerous tissues (Figure 1C).

### MLPH affects prognosis in PAAD patients

The study of the correlation between MLPH expression and prognosis was performed using the Kaplan-Meier Plotter database, which showed that MLPH was significantly associated with prognosis in PAAD patients (Figure 1D). In addition, we analyzed the relationship between MLPH expression and clinical characteristics. The results showed that the mRNA expression of MLPH was significantly higher in high grade patients than in low grade patients, and the expression of MLPH was significantly correlated with histologic grade (Figure 1E).

### MLPH promotes cell proliferation

**Figure 2:**
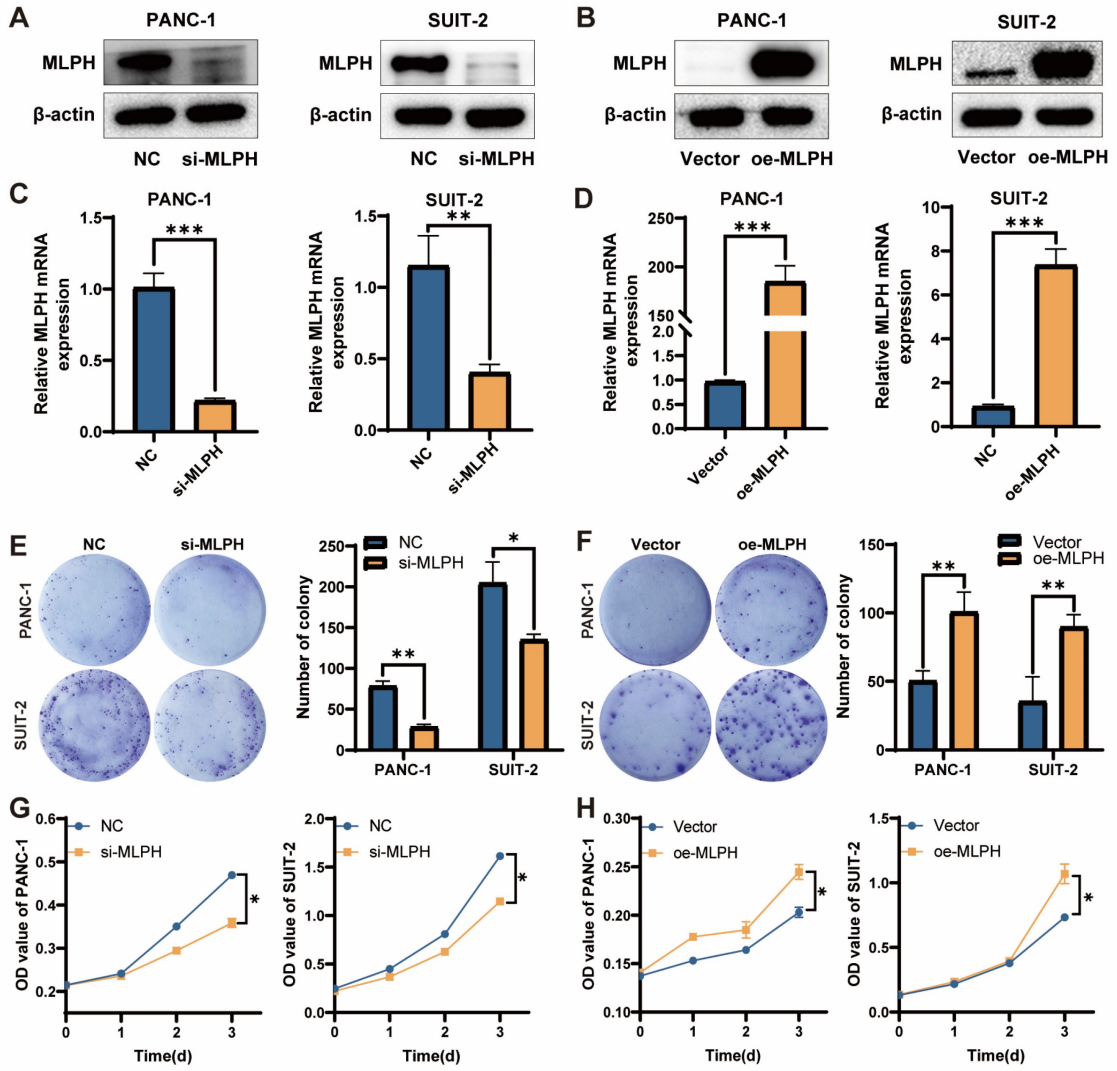
** MLPH enhances the proliferation of PAAD cells.** (A, B) Protein levels of MLPH in cells were detected by western blotting after MLPH knockdown and overexpression, respectively. (C, D) The mRNA levels of MLPH in cells were detected by real-time fluorescence quantitative PCR after MLPH knockdown and overexpression, respectively. (E, F) Quantitative statistics of the number of colonies and the number of colonies of cells detected by clone formation assay after MLPH knockdown and overexpression are shown, respectively. (G, H) Proliferative capacity of cells was detected by CCK-8 assay after MLPH knockdown and overexpression, respectively. (si-MLPH group vs. negative control group; oe-MLPH group vs. Vector group, *P < 0.05, ** *P* < 0.01, *** *P* < 0.001).

MLPH expression in the PANC-1 and SUIT-2 cells was inhibited using si-MLPH (Figure 2A, C), and was overexpressed using an MLPH overexpression vector (Figure 2B, D). To investigate the effect of MLPH on cells, we performed phenotypic validation. In the colony formation assay, the colony number in the cells was significantly reduced after MLPH knockdown, compared to the negative control group (Figure 2E), whereas the colony number in the cells was significantly increased after MLPH overexpression, compared to the Vector group (Figure 2F). In the CCK-8 assay, the si-MLPH-treated cells had a significantly lower proliferation rate than the NC group (Figure 2G). The MLPH-overexpressing cells grew significantly faster than those in the Vector group (Figure 2H).

### MLPH enhances PAAD cell migration and invasion

**Figure 3:**
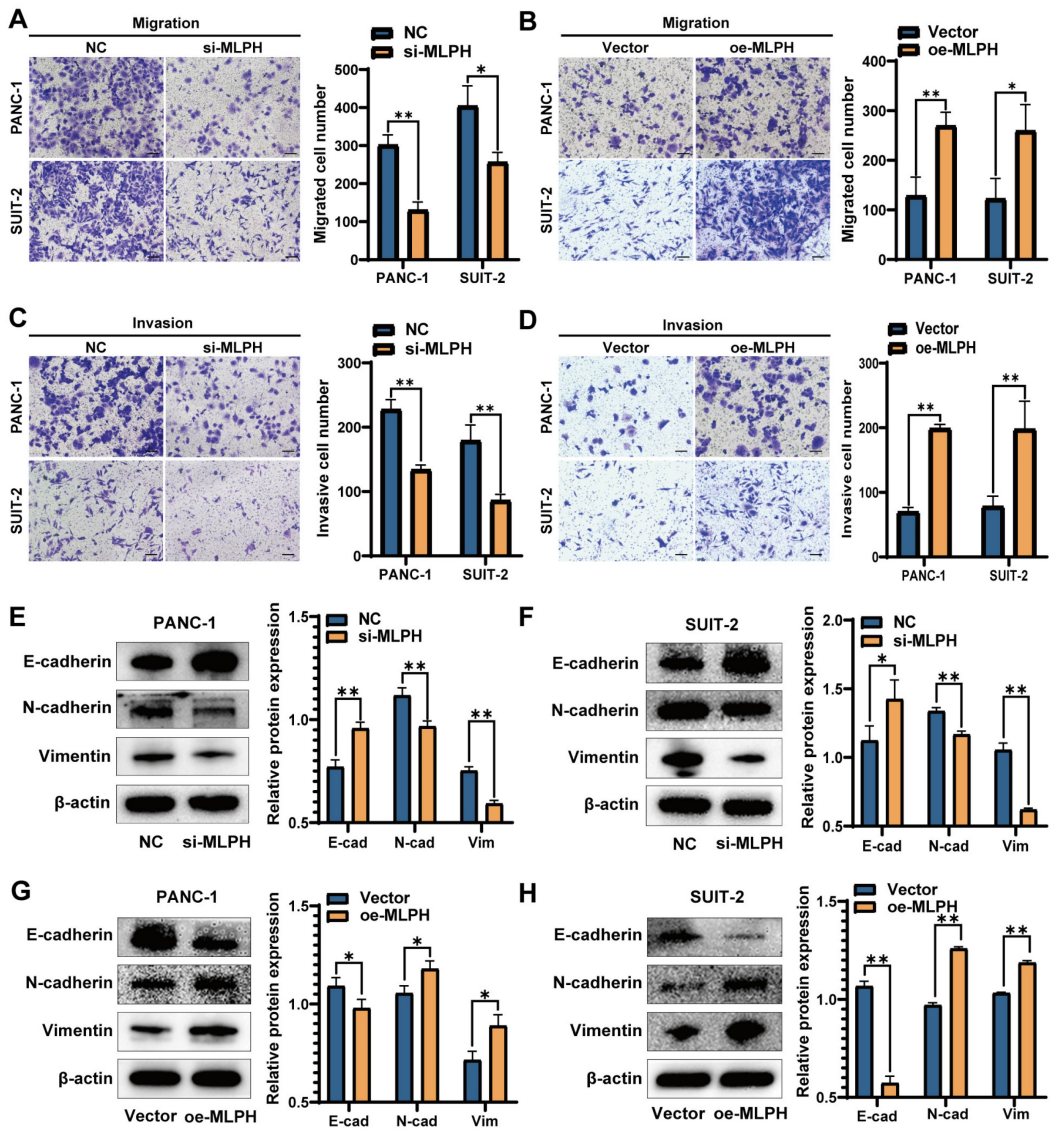
** MLPH promotes the migration and invasive capacity of PAAD cells.** (A) After the knockdown of MLPH, the migratory ability of the cells was examined through chambers without the addition of matrix gel. (B) After overexpression of MLPH, the migratory capacity of the cells was examined through chambers without the addition of matrix gel. (C) After the knockdown of MLPH, the invasive ability of the cells was tested by the addition of matrix gel to the chambers. (D) After overexpression of MLPH, the invasive capacity of the cells was detected by adding matrix gel to the chambers. (E, F) After overexpression of MLPH, the protein expression of EMT markers (E-cadherin, N-cadherin, and vimentin) were measured by western blotting. (G, H) After the knockdown of MLPH, the protein expression of EMT markers were measured by western blotting. (si-MLPH group vs. negative control group; oe-MLPH group vs. Vector group; The scale bar is 100 μm, *P < 0.05, ** *P* < 0.01).

The Transwell migration assay demonstrated that MLPH knockdown inhibited migration as compared to the control (Figure 3A). The Transwell invasion assay demonstrated that MLPH knockdown decreased the cell invasion rate, compared to the control (Figure 3C). Furthermore, the MLPH-overexpressing cells demonstrated increased migration (Figure 3B) and invasion (Figure 3D) rates, compared to the Vector group.

### MLPH activates PAAD cell EMT and the PI3K-AKT pathway

The experiments on cell phenotyping revealed that MLPH was closely linked to cell proliferation, migration, and invasion. Western blot detection of changes in EMT-related active molecules and markers participating in PAAD cells revealed that MLPH knockdown significantly decreased N-cadherin and vimentin, and increased E-cadherin levels as compared with the NC group (Figure 3E, F). MLPH overexpression increased the N-cadherin and vimentin, and decreased the E-cadherin levels, compared to the Vector group (Figure 3G, H).

### MLPH regulates PAAD cell EMT via the PI3K-AKT pathway

**Figure 4:**
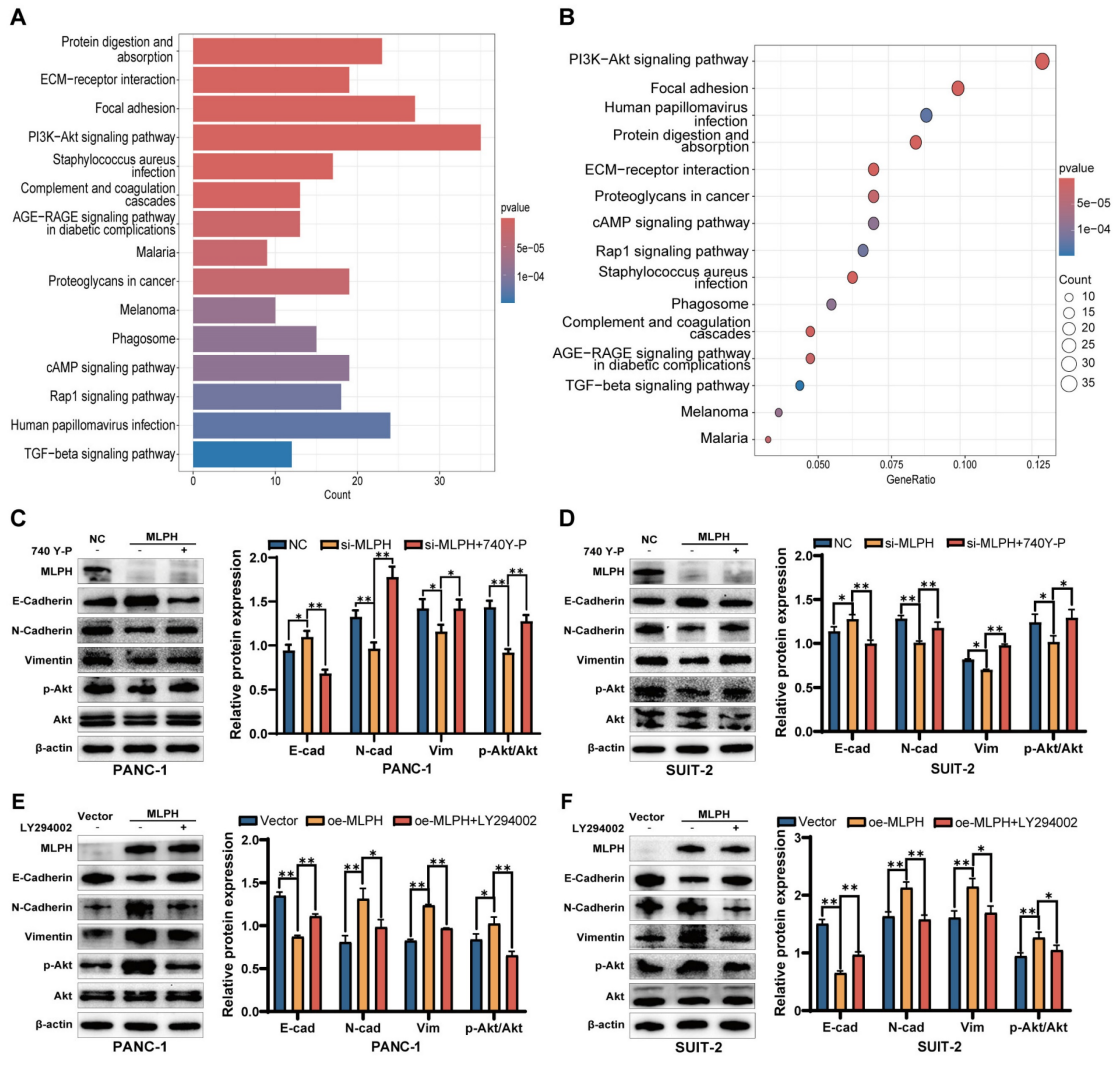
** MLPH promotes EMT in PAAD cells through the PI3K-AKT signaling pathway.** (A, B) KEGG Pathway enrichment analysis by KEGG based on TCGA database. (C, D) Relative protein levels of MLPH, E-cadherin, N-cadherin, Vimentin, p-Akt, and Akt in cells after treatment with or without 740Y-P and the quantitative statistics of protein expression. (*P < 0.05, ** *P* < 0.01). (E, F) Relative protein levels of MLPH, E-cadherin, N-cadherin, Vimentin, p-Akt, and Akt in cells after treatment with or without LY294002 and the quantitative statistics of protein expression. (*P < 0.05, ** *P* < 0.01).

The KEGG enrichment analysis demonstrated that the molecular mechanism by which MLPH affected PAAD was significantly associated with the PI3K-AKT pathway (Figure 4A, B). We treated MLPH-silenced cells with the PI3K-AKT pathway activator 740Y-P to ascertain the role of PI3K-AKT pathway activation in MLPH-mediated EMT. The 740Y-P reversed the expression changes of E-cadherin, N-cadherin, and vimentin expression changes (Figure 4C, D). Then, we treated the MLPH-overexpressing cells with the PI3K-AKT pathway inhibitor LY294002, which significantly inhibited the expression levels of E-cadherin, N-cadherin, and vimentin (Figure 4E, F). The results indicated that MLPH controls pancreatic cancer cell EMT by activating the PI3K-AKT pathway.

### MLPH promotes tumor progression *in vivo*

**Figure 5:**
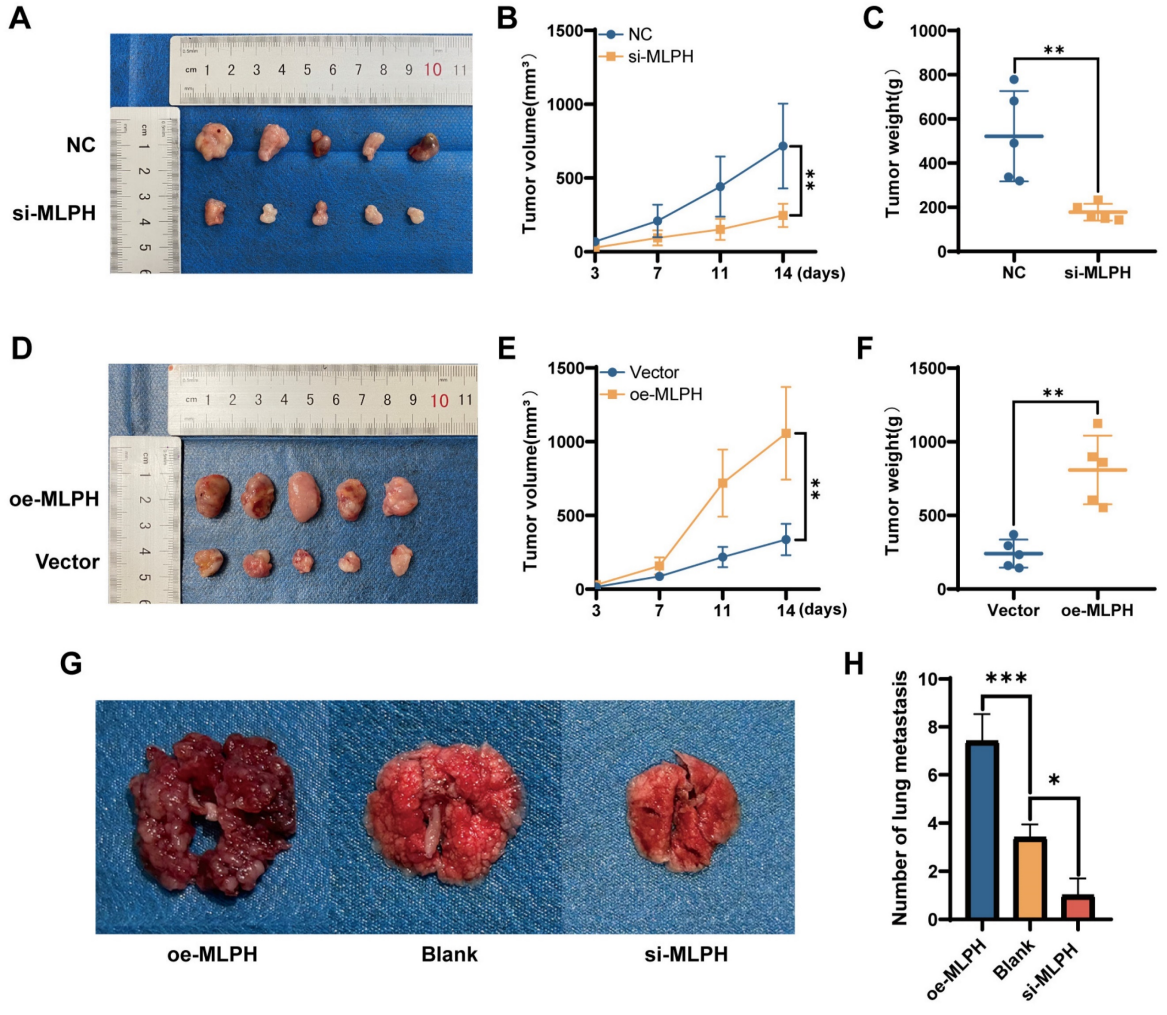
** MLPH promotes tumor progression *in vivo*.** (A) Subcutaneous tumor formation in nude mice after MLPH knockdown. (B) The tumor growth curves of subcutaneous tumor in nude mice after MLPH knockdown. (C) Subcutaneous tumor weight in nude mice after MLPH knockdown. (D) Subcutaneous tumor formation in nude mice after MLPH overexpression. (E) The tumor growth curves of subcutaneous tumor in nude mice after MLPH overexpression. (F) Subcutaneous tumor weight in nude mice after MLPH overexpression. (G) The general appearance of lung metastasis. (H) The number of lung metastasis nodules. (*P < 0.05, ** *P* < 0.01, *** *P* < 0.001).

To validate the effect of MLPH on pancreatic cancer *in vivo*, we constructed a subcutaneous xenograft model using SUIT-2 cells after MLPH knockdown and overexpression (n =5/group). MLPH knockdown inhibited tumor growth compared to the NC group (Figure 5A), the volume and weight of the tumor confirmed this equally (Figure 5B, C). On the contrary, MLPH overexpression promoted tumor growth compared to the Vector group (Figure 5D), and it is also consistent with tumor volume and weight measurements (Figure 5E, F). To thoroughly validate the impact of MLPH on tumor metastasis *in vivo*, we constructed a nude mouse tail vein lung metastasis model. After 4 weeks, the number of lung metastatic nodules was significantly reduced in the MLPH knockdown group compared with the blank group, and the number of lung metastatic nodules was significantly increased in the MLPH overexpression group (Figure 5G, H). These results suggest that MLPH promotes the growth and metastasis of pancreatic cancer cells *in vivo*.

## Discussion

PAAD is a common malignant tumor globally. Distant metastasis occurs in >60% of patients within 24 months after surgery. So, tumor cell metastasis presents a major clinical challenge in tumor treatment.

EMT is important in tumor cell metastasis^11^, ^31^. During EMT, epithelial cells lose their polarization properties and stable cell adhesion, and gain a mesenchymal cell phenotype, thereby promoting tumor invasion and metastasis^32^, ^33^. Furthermore, EMT is also closely linked to PAAD migration and invasion progression. Thus, regulating EMT is crucial to control pancreatic cancer cell migration and invasion.

It was reported that MLPH correlates with the progression of various tumors, and has become a prognostic indicator and treatment target for a variety of tumors. In rectal cancer, high MLPH expression in patients was associated with poorer preoperative radiotherapy response and lower survival^22^. In melanoma, MLPH blocker inhibits melanoma cell motility and invasion^17^. Furthermore, in prostate cancer studies, MLPH influences tumor progression by upregulating EMT^20^. However, the specific mechanism and role of MLPH in PAAD have not been studied. Accordingly, we speculated whether MLPH is differentially expressed between PAAD and noncancerous tissues, impacts patient prognosis, and is associated with PAAD invasion and migration.

We examined TCGA gene expression data, which demonstrated that PAAD tissues had higher MLPH expression than noncancerous pancreatic tissues. Subsequently, we analyzed HPA immunohistochemical samples and verified the earlier gene expression analysis results. To explore the correlation between MLPH expression and prognosis, we performed prognostic analyses through the Kaplan-Meier Plotter database. The results showed that MLPH was significantly associated with prognosis in pancreatic cancer patients. In a clinical correlation analysis based on TCGA-PAAD data, we found that MLPH was significantly associated with histologic grade. MLPH is highly expressed in high-grade tumors, suggesting that it is associated with tumor prognosis and may be a potential target for pancreatic cancer therapy.

Subsequently, we explored the role of MLPH in PAAD proliferation, invasion, and migration by cell function experiments and western blot. We constructed MLPH knockdown and MLPH overexpression cell lines for *in vivo* and *in vitro* validation to examine the potential MLPH biological functions in PAAD. MLPH knockdown decreased PAAD cell proliferation, migration, and invasion ability, increased E-cadherin expression, and decreased N-cadherin and vimentin expression. The results suggested that MLPH knockdown inhibited EMT in PAAD. Contrastingly, MLPH overexpression enhanced PAAD cell ability to proliferate, migrate, and invade, decreased E-cadherin expression, and decreased N-cadherin and vimentin expression, which increased EMT in PAAD cell. The findings all suggested that MLPH is an important EMT regulator in PAAD cells.

We also performed KEGG enrichment analysis based on the TCGA data. Several studies reported PI3K-AKT signaling pathway activation is closely associated with tumor cell invasion and metastasis^34^. In PAAD, genes such as CPA4, PGAM1, and PAR4 contribute to PAAD metastasis through the PI3K-AKT pathway 35-37. In the present study, the KEGG enrichment analysis demonstrated that MLPH expression in pancreatic cancer correlated significantly with the PI3K-AKT pathway, suggesting that MLPH might regulate EMT in PAAD through the PI3K-AKT pathway. Subsequently, we confirmed that MLPH expression increased AKT expression levels, which suggested that MLPH activated the PI3K-AKT pathway in PAAD cells. To ascertain whether PI3K-AKT pathway activation is vital to MLPH-mediated EMT, MLPH knockdown cells and MLPH overexpression cells were treated with activator and inhibitor of PI3K-AKT pathway, respectively, and replication experiments were performed. The experiments demonstrated that the activator and inhibitor reversed the effects of MLPH downregulation and overexpression, respectively, on the PAAD cells.

In conclusion, we demonstrated that MLPH mediates the PI3K-AKT pathway to control EMT in PAAD cells. However, how MLPH controls the PI3K-AKT pathway, and the specific and immediate targets of MLPH regulation of the PI3K-AKT pathway, have not been clarified. In summary, we believe that MLPH can promote PAAD cell EMT by activating the PI3K-AKT pathway, thereby promoting cell invasion and migration. Therefore, it could be a potential prognostic factor and target for blocking PAAD metastasis.

## Abbreviations

PAAD: Pancreatic adenocarcinoma
MLPH: Melanophilin
EMT: Epithelial—mesenchymal transition
KEGG: Kyoto Encyclopedia of Genes and Genomes
TCGA: The Cancer Genome Atlas
GEPIA 2: the Gene Expression Profiling Interaction Analysis 2
HPA: Human Protein Atlas databases
LY294002: a PI3K-AKT pathway inhibitor
740Y-P: a PI3K-AKT pathway activator
